# Nanopore‐Based Protein Deceleration and Sensing Using Graphene/Si_3_N_4_ Dual Membrane Cavity

**DOI:** 10.1002/advs.76099

**Published:** 2026-06-11

**Authors:** Yubin Cao, Junzhou He, Wei Si

**Affiliations:** ^1^ Jiangsu Key Laboratory for Design and Manufacturing of Precision Medicine Equipment School of Mechanical Engineering Southeast University Nanjing China

**Keywords:** cavity structure, deceleration, nanopore, protein sequencing, translocation rate

## Abstract

Sequencing of protein with nanopores has emerged as a powerful tool offering rapid readout, high accuracy, low cost, and portability. There is an urgent need to improve the sensing accuracy of nanopores to effectively realize protein sequencing. However, controlling protein translocation rates remains a significant challenge. This study designed a graphene/Si_3_N_4_ dual membrane cavity system to explore potential solutions for enhancing protein sequencing accuracy and efficiency. Charge regulation and cavity structure were employed to slow down the translocation rate of peptides in the Si_3_N_4_ nanopore. The proposed Si_3_N_4_ toroidal cavity structure successfully reduces peptide translocation rates by introducing physical steric hindrance and enhancing van der Waals adsorption. This structure not only substantially prolongs peptide residence time in the sensing region but also maintains excellent signal resolution, offering a potential approach to address the high bandwidth demands in single‐molecule protein sequencing. The findings contribute to achieving high‐resolution, high‐throughput protein sequencing and advance the field of proteomics.

## Graphical abstract

A graphene/Si_3_N_4_ dual membrane cavity system achieves multi‐stage protein deceleration through spatial geometry. Multi‐level current traces provide high‐resolution spatial localization and early warning signals.

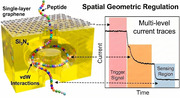

## 1 Introduction

Proteins serve as vital biological macromolecules in living organisms, playing critical roles in human physiology [1]. The function or dysfunction of proteins largely depends on their primary structure, the amino acid sequence, which is essential for determining spatial structure and function [2]. However, protein sequencing is analytically more complex, with 20 canonical as well as hundreds of non‐proteinogenic amino acids, including many post‐translational modifications [3, 4, 5]. Subtle changes to this amino acid sequence may cause a dramatic loss of bioactivity and may consequently cause relevant diseases [6]. While Edman degradation [7] and Mass Spectrometry [8, 9] are mature technologies for analyzing protein sequences and structures, these ensemble‐averaging methods struggle to capture instantaneous conformational changes and stochastic dynamic behaviors at the single‐molecule level. Consequently, they possess inherent limitations in revealing the details of protein folding and function [3].

To overcome current technological bottlenecks, various emerging single‐molecule techniques have been developed, including tunneling currents [10, 11, 12], optical tweezers [13], fluorescence fingerprinting [14, 15], and nanopore sequencing [16, 17]. These methods aim to achieve sequencing or identification of single proteins [18]. Among them, nanopore sequencing has garnered significant attention due to its label‐free, amplification‐free, and high‐throughput characteristics [19, 20]. Its fundamental principle involves utilizing an applied electric field to drive individual molecules through nanoscale channels [21, 22]. Ionic current fluctuations could be observed as molecules pass through a single nanochannel or nanopore [23, 24]. Subsequently, the ionic current changes are decoded using base‐calling algorithms to characterize the molecule and even determine the composition of the molecule [25, 26]. This makes it an ideal potential technology for protein sequencing [27].

In recent years, nanopore‐based single‐molecule sequencing has been developed rapidly [28], with increasingly mature fabrication processes for nanopores of various materials [29, 30]. However, this technology faces a core contradiction, to precisely control peptide translocation and minimize diffusion and regression phenomena, a high driving voltage is typically required. Paradoxically, this significantly shortens the peptide's dwell time within the pore. Under electric fields, protein molecules often pass through the nanopore at rate exceeding the temporal resolution limits of existing detection equipment for distinguishing single amino acids [31, 32, 33]. Achieving an optimal translocation rate remains a major challenge. Therefore, enabling proteins to pass through nanopores at an appropriate rate is an urgent problem to solve for more sensitive and precise protein sequencing.

To date, researchers have proposed various strategies to control the translocation rate of biomolecules to improve sensing precision. These include altering nanopore diameter [34, 35], material [36, 37, 38], and charge [39]. Other methods involve adjusting solution viscosity [40] or temperature [41]. However, adjusting viscosity may introduce complex chemical background noise, while changing temperature can lead to biomolecule inactivation. Alternative approaches, such as magnetic tweezers or atomic force microscopy control [42] can also effectively regulate translocation rate, but they introduce non‐native complex substances that interfere with observations and affect signal quality. Consequently, a simple and effective method is needed to slow down proteins without complicating the sensing environment.

In this work, we proposed a proof‐of‐concept method that realize nanopore‐based protein deceleration and sensing using graphene/Si_3_N_4_ dual membrane cavity. Using molecular dynamics (MD) simulations, we investigated the possibility of using the designed cavity structure to slow down the translocation rate of peptide. We found that simply forming cavity channel or adjusting entrance charges does not effectively solve the deceleration problem in the critical sensing region. Conversely, the eccentric structure can effectively reduce the translocation rate of peptide in the Si_3_N_4_ nanopore region by enhancing the interaction energy with the sidewalls. Furthermore, the proposed Si_3_N_4_ roundabout in the cavity can significantly slow down the translocation rate of peptide by introducing physical steric hindrance and enhancing the interaction energy. The designed structure in this work not only substantially prolongs the dwell time of peptide in the sensing region but also maintains good signal resolution, providing an another potential solution for the high‐bandwidth demands of protein sequencing. These results contribute to the realization of high‐resolution, high‐throughput protein sequencing and promote the development of proteomics.

## 2 Results and Discussion

**FIGURE 1:**
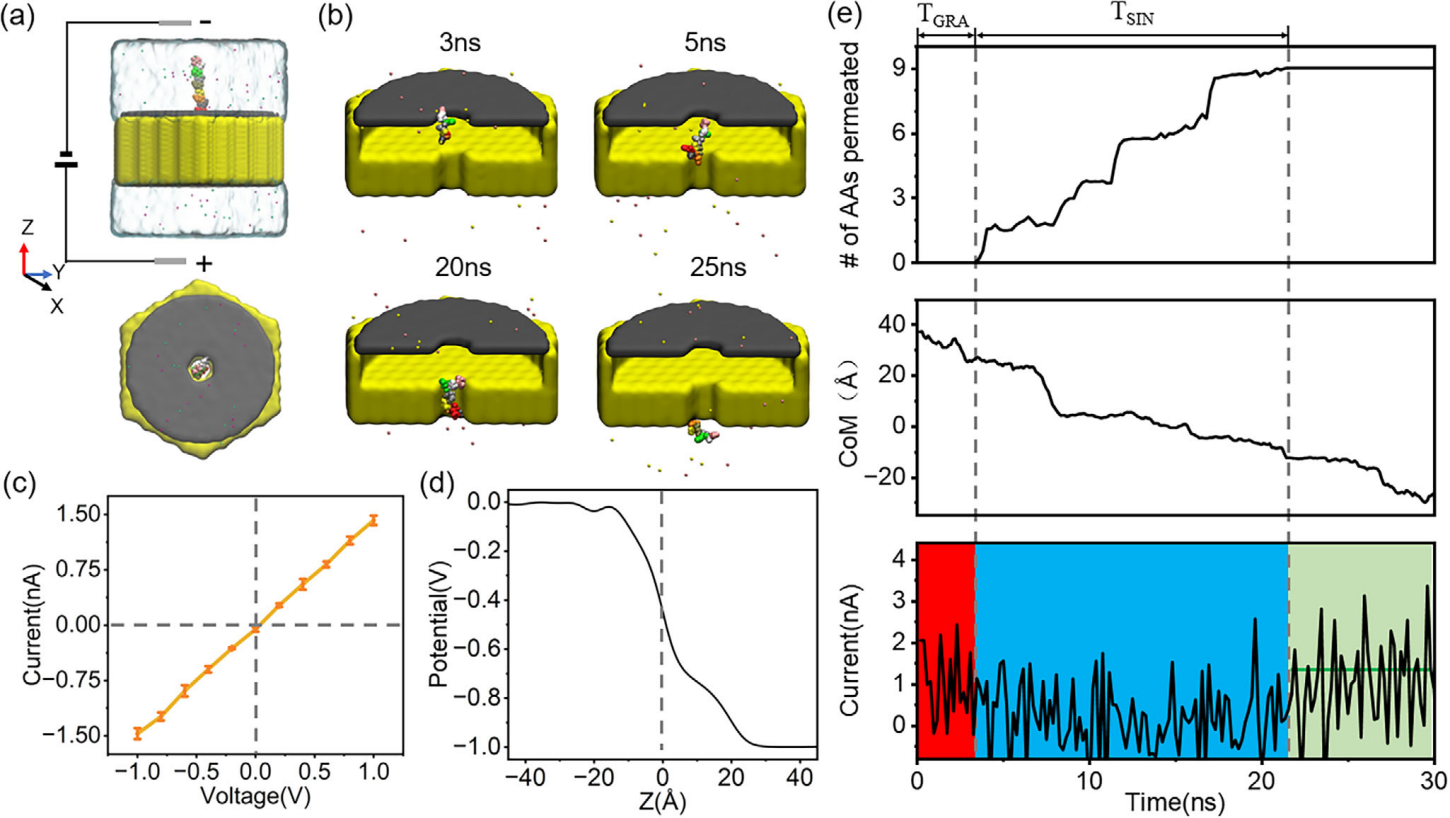
Simulations of peptide translocation through the coaxially aligned nanopores at the center of the graphene/Si_3_N_4_ dual membrane cavity structure. (a) Side and top view of the simulation system. The Si_3_N_4_ is shown in yellow, with the nanopore boundary highlighted in red. The graphene is shown in gray. The peptide consisting of nine amino acids is shown using vdW spheres. The cyan transparent surface represents the liquid solution. Potassium and chloride ions are displayed as small magenta and green spheres, respectively, distributed throughout the solution. (b) A series of microscopic configurations of peptide translocation through the cavity structure vs. time, which observed as the peptide moves electrophoretically from the *cis* side to *trans* side. (c) *I–V* characteristics of the system obtained from simulations of the bare pores. The applied biases range from −1 to 1 V, incremented by 0.2 V. Error bars represent the standard deviation. (d) The electrostatic potential along the coaxially aligned nanopore axis under an electrical field of 10.21 mV/Å. The dashed line indicates the position of the system center along the z‐axis. (e) The number of protein residues permeated through the Si_3_N_4_ nanopore, the center of mass position of peptide along *z* axis and the ionic current trace vs. time during the entire translocation process of peptide through the cavity structure, respectively. For the current trace, the red and blue regions correspond to the peptide translocation through the graphene and Si_3_N_4_ nanopores; while the green region corresponds to that the peptide has fully exited the Si_3_N_4_ nanopore.

In this work, we first simulated peptide translocation through the coaxially aligned nanopores at the center of the graphene/Si_3_N_4_ dual membrane cavity structure (Figure 1). In this simulation, both the graphene and Si_3_N_4_ nanopores are aligned along the central axis, creating a direct vertical path (Figure 1a). Once the negatively charged peptide is captured, it is driven through the nanopores by the electrophoretic force generated by the external electric field. The peptide undergoes a complete migration process, it is firstly captured by the graphene nanopore, then it enters the cavity and finally exits from the Si_3_N_4_ nanopore (Figure 1b). The peptide translocated through the graphene nanopore in approximately 4 ns. After passing through the graphene nanopore, as both the graphene and Si_3_N_4_ nanopores are coaxially aligned in the middle of the cavity, the peptide then quickly entered the Si_3_N_4_ nanopore, ultimately completing its translocation through the Si_3_N_4_ nanopore in about 24 ns. Movie S1 shows the simulation trajectory of the whole process for translocation. Figure 1c describes the *I*–*V* characteristic relationship of the system under different voltages. For this system, we observe a linear relationship between the bias voltage and the ionic current, indicating compliance with Ohm's law. Strong electric fields force cations (K^+^) and anions (Cl^−^) to move in opposite directions, thereby generating an open‐pore current. Additionally, we performed additional simulations using the same system but for different electric fields, see Figure S1. Furthermore, the two‐dimensional electrostatic potential distribution along the pore axis indicates that the voltage drop is primarily concentrated at the nanopore region (Figure 1d), serving as the primary driving force for the quick and successful translocation of peptide through the coaxially aligned nanopores at the center of the graphene/Si_3_N_4_ dual membrane cavity structure.

The above simulation results indicate that the peptide translocation through the nanopore rapidly under the strong external electric force. As the peptide is captured and translocating through the nanopore, it generates distinct ionic current blockade signals (Figure 1e) during its permeating process. Here, to separately investigate the translocation process and dynamics of peptide through the graphene nanopore, cavity and Si_3_N_4_ nanopore in sequence, we defined the time required for the peptide to translocate through the graphene nanopore as *T_GRA_*, the time for peptide translocation through the cavity channel as *T_CAV_*, and the time for peptide translocation through the Si_3_N_4_ nanopore as *T_SIN_*. Since the graphene and Si_3_N_4_ nanopores are coaxially aligned and located at the center of cavity structure, the peptide enters the Si_3_N_4_ nanopore immediately after translocation through the graphene nanopore, the total translocation time is then defined as the sum of the times required to translocation through both pores. Statistical analysis of peptide residue number, center of mass (CoM) positions, and ionic current trace vs. time are performed. The results show that two current levels are observed sequentially due to peptide residing in the graphene nanopore and Si_3_N_4_ nanopore respectively, and finally the current recovers to the open‐pore current after the peptide fully translocated through the cavity structure. Similar results for other three successful peptide translocation cases through the cavity structure are presented in Figure S2. Interestingly, for almost all the simulations, we observed extremely rapid translocation of the peptide through the entire system, such short dwell times actually prevent collection of sufficient current signal data to distinguish amino acid types, indicating that effective deceleration of peptide in the cavity structure is very essential for nanopore based protein detection and sequencing.

**FIGURE 2:**
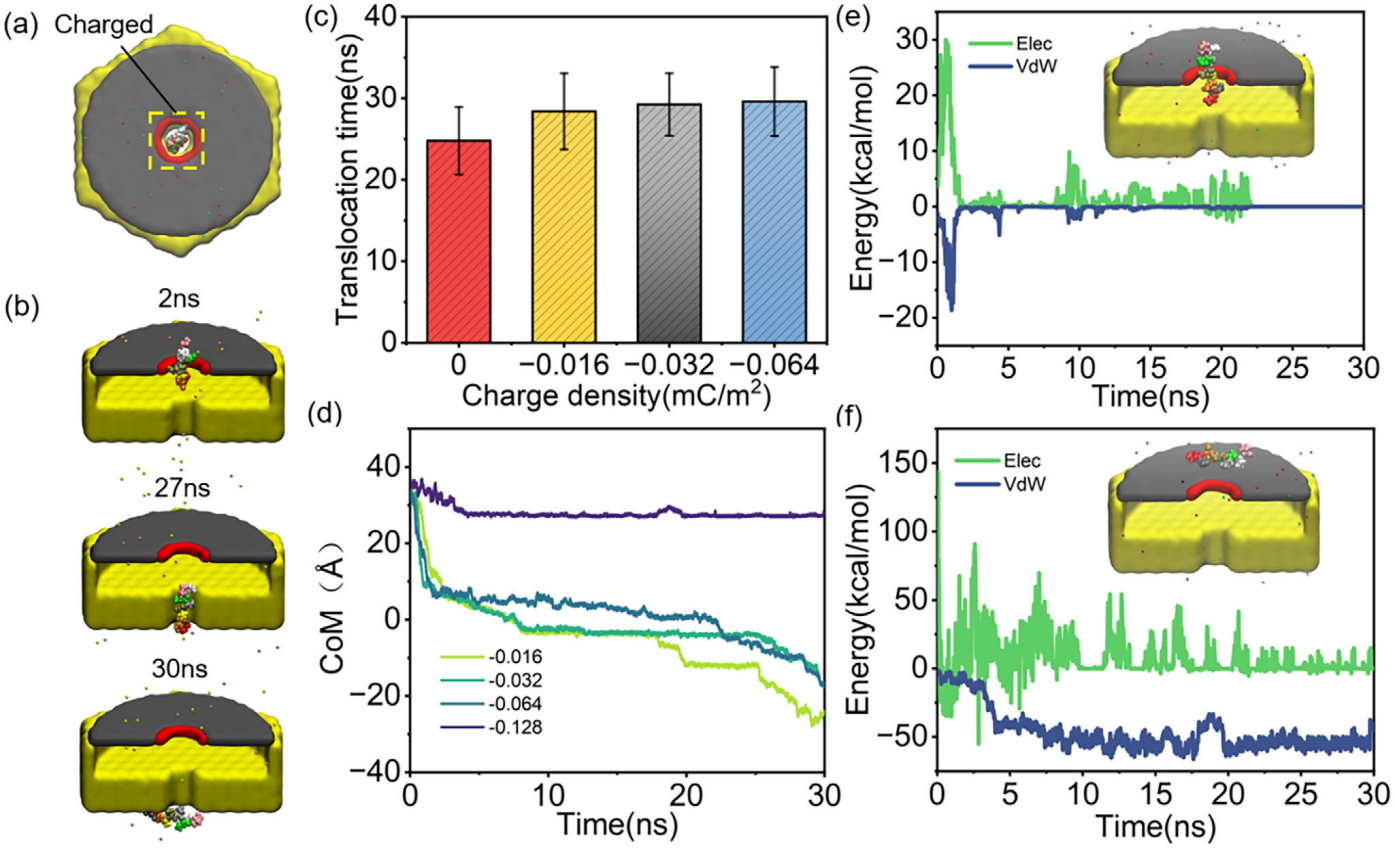
Simulations of peptide translocation through the coaxially aligned nanopores at the center of the graphene/Si_3_N_4_ dual membrane cavity structure with charged graphene nanopore. (a) Top view of the simulation system. The coloring method for all the materials in the system is same as that used for Figure 1. The yellow dashed highlights the red graphene nanopore, indicating that the surface charge density of graphene nanopore is tuned in the simulations. (b) A series of microscopic configurations of peptide translocation through the cavity structure vs. time, were obtained with the graphene nanopore surface charge density of − 0.016*mC*/*m* ^2^. (c) Translocation time of the peptide through the cavity structure under different surface charge densities. (d) The center of mass position of the peptide along z axis vs. time for different surface charge densities. (e) The interaction energy between the peptide and graphene membrane vs. time with surface charge density of − 0.016*mC*/*m* ^2^. Electrostatic interaction energy and van der Waals interaction energy are represented by green and purple solid lines, respectively. (f) Similar to panel e but for the surface charge density of − 0.128*mC*/*m* ^2^.

In a typical nanopore sensor, a charged nanopore would reduce the translocation speed of molecules that carry like charges due to the repelling force between the nanopore and molecules as well as the electroosmotic force generated in the charged nanopore. Based on the simulation system shown in Figure 1, charges are assigned to the graphene nanopore to study the effect of surface charge density (*σ*) on the peptide translocation dynamics through the cavity structure (Figure 2a). Figure 2b illustrates the typical migration conformation of the peptide for the surface charge density of − 0.016 *mC*/*m* ^2^. Movie S2 shows the simulation trajectory of the whole process for translocation. Additional simulation results regarding the number of protein residues permeated through the Si_3_N_4_ nanopore, center of mass position of the peptide, ionic current trace are provided in Figure S3. Figure 2c shows the time required for the peptide translocation through the system under different surface charge densities. The results indicate that compared to the uncharged condition, a negatively charged graphene nanopore does increase the total translocation time for peptide translocating through the cavity structure, and this increment is primarily attributed to the time translocation through the graphene nanopore (Figure S4) with different charges.

Furthermore, as shown in Figure 2d, the peptide can only successfully translocate through graphene nanopores with surface charge density magnitude smaller than 0.128 *mC*/*m* ^2^, further increasing the amplitude of surface charge density (for example, *σ* = − 0.128 *mC*/*m* ^2^), prevents the peptide being captured by the nanopore and keeps the peptide outside the nanopore. To unveil the mechanism, we analyzed the interaction energy between the peptide and graphene membrane. As shown in Figure 2e,f, *σ* = − 0.016 *mC*/*m* ^2^, the peptide initially approaches the negatively charged graphene nanopore driven by the electric force. During this initial process, the van der Waals energy and the electrostatic energy are opposite as the graphene surface attracts the peptide (vdW force dominates) and the charged nanopore repels the peptide with like charges (electrostatic repelling force dominates). Subsequently, as the peptide approaches the graphene surface, the strong electric field force and van der Waals force overcome the electrostatic repulsion, successfully making the peptide be trapped by the nanopore. The bias voltage serves as the primary driver, helping the peptide overcome the initial energy barrier. However, when *σ* = − 0.128 *mC*/*m* ^2^, the initial electrostatic repulsion energy is approximately 150 kcal/mol, far exceeding the vdW attraction. This creates an energy barrier higher than the energy provided by the electrophoretic drive, resulting in capture failure. However, once the peptide is captured by the graphene nanopore, it is quickly captured by and translocates through the Si_3_N_4_ nanopore. In summary, regulating the charge of the graphene nanopore can slightly reduce the translocations rate through the graphene pore itself but cannot effectively decelerate the peptide through the Si_3_N_4_ nanopore in the cavity structure.

**FIGURE 3:**
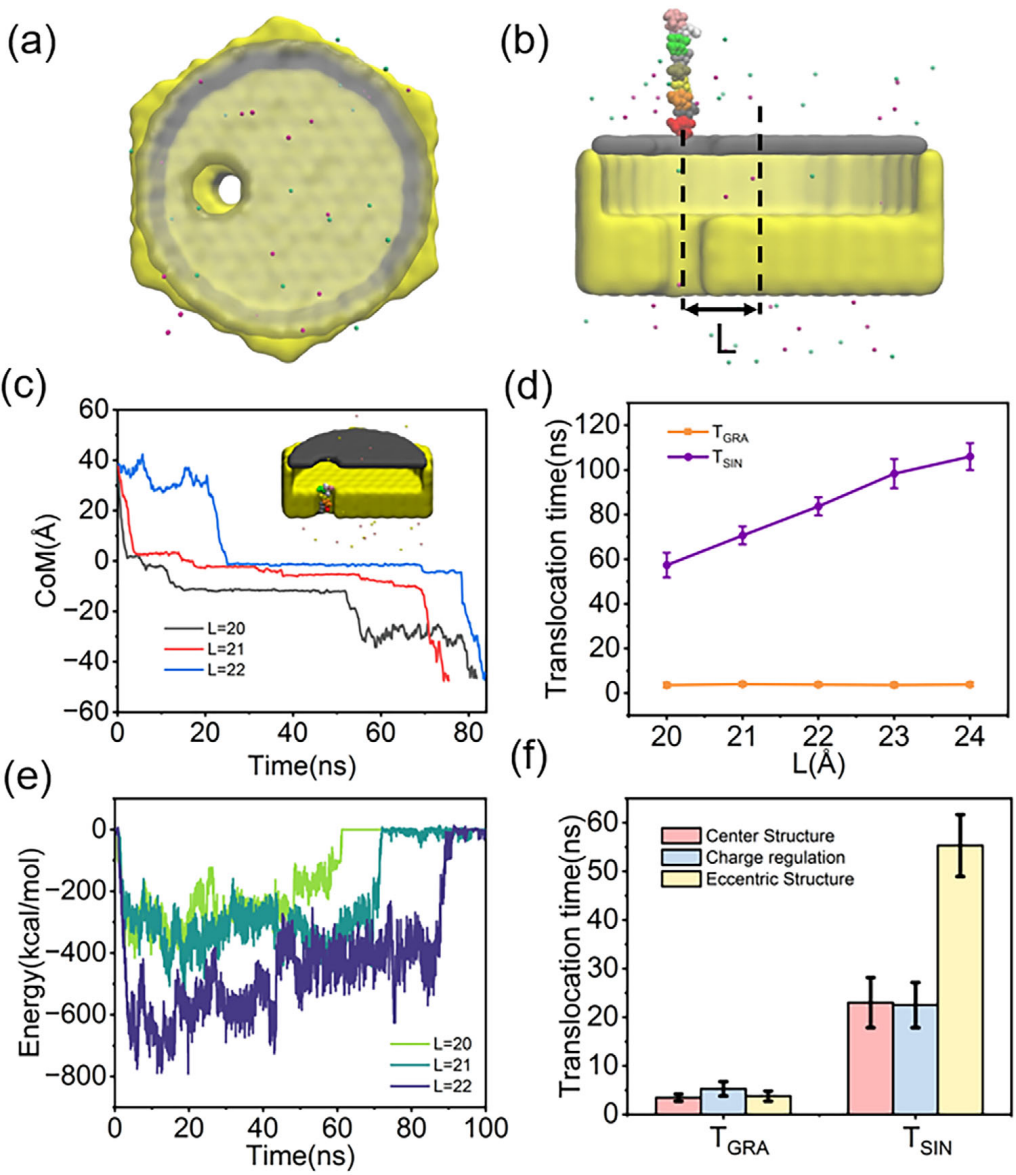
Simulations of peptide translocation through the coaxially aligned nanopores displaced from the center of the graphene/Si_3_N_4_ dual membrane cavity structure. (a) Top view of the simulation system (graphene layer transparent for clarity). The coloring method for all the materials in the system is same as that used for Figure 1. (b) Side view of the simulation system. *L* denotes the distance between the center of nanopore and the center of cavity structure. (c) The center of mass position of the peptide along z axis vs. time for different *L* values; the insert figure shows a typical example of the microscopic conformation of peptide passing through the cavity structure. (d) The translocation times required for the peptide passing through the separate graphene and Si_3_N_4_ nanopores for different *L* values, respectively. (e) The interaction energy between the peptide and Si_3_N_4_ membrane vs. time for different *L* values. (f) Comparison of the translocation time required for the peptide passing through the cavity structures.

To slow down the translocation rate of the peptide through the Si_3_N_4_ nanopore in the cavity structure, we attempted to offset the positions of the graphene and Si_3_N_4_ nanopores to one side of the system, forming a graphene/Si_3_N_4_ dual membrane cavity structure with the coaxially aligned nanopores displaced from the center of the cavity structure, as shown in Figure 3a,b. This design aims to investigate whether positioning the Si_3_N_4_ nanopore closer to the sidewalls affects the peptide's translocation rate. Movie S3 shows the simulation trajectory of the whole process for translocation. For the eccentric nanopores, the peptide could also successfully translocate through them, see the *z* component of the peptide's CoM shown in Figure 3c. The corresponding amino acid residue number of peptide permeating through the nanopore is shown in Figure S5. Furthermore, as the distance (*L*) between the nanopore and the cavity center increases, the time required for the peptide to translocate through the Si_3_N_4_ nanopore increases significantly (Figure 3d). Interaction energy analysis reveals that as the distance *L* increases, the interaction energy between the peptide and the Si_3_N_4_ strengthens (Figure 3e). This indicates that the adsorption capacity of the Si_3_N_4_ for the peptide becomes stronger, effectively slowing down its translocation rate. Multiple sets of data on the interaction energy are provided in Figure S6. Figure 3f clearly compares the translocation time through the graphene and Si_3_N_4_ nanopores across the three systems shown in Figures 1, 2, 3. The results show that while the surface charge on graphene nanopore only slightly increases the translocation time, by placing the coaxially aligned nanopores away from the cavity center significantly increases the translocation time through the Si_3_N_4_ nanopore.

**FIGURE 4:**
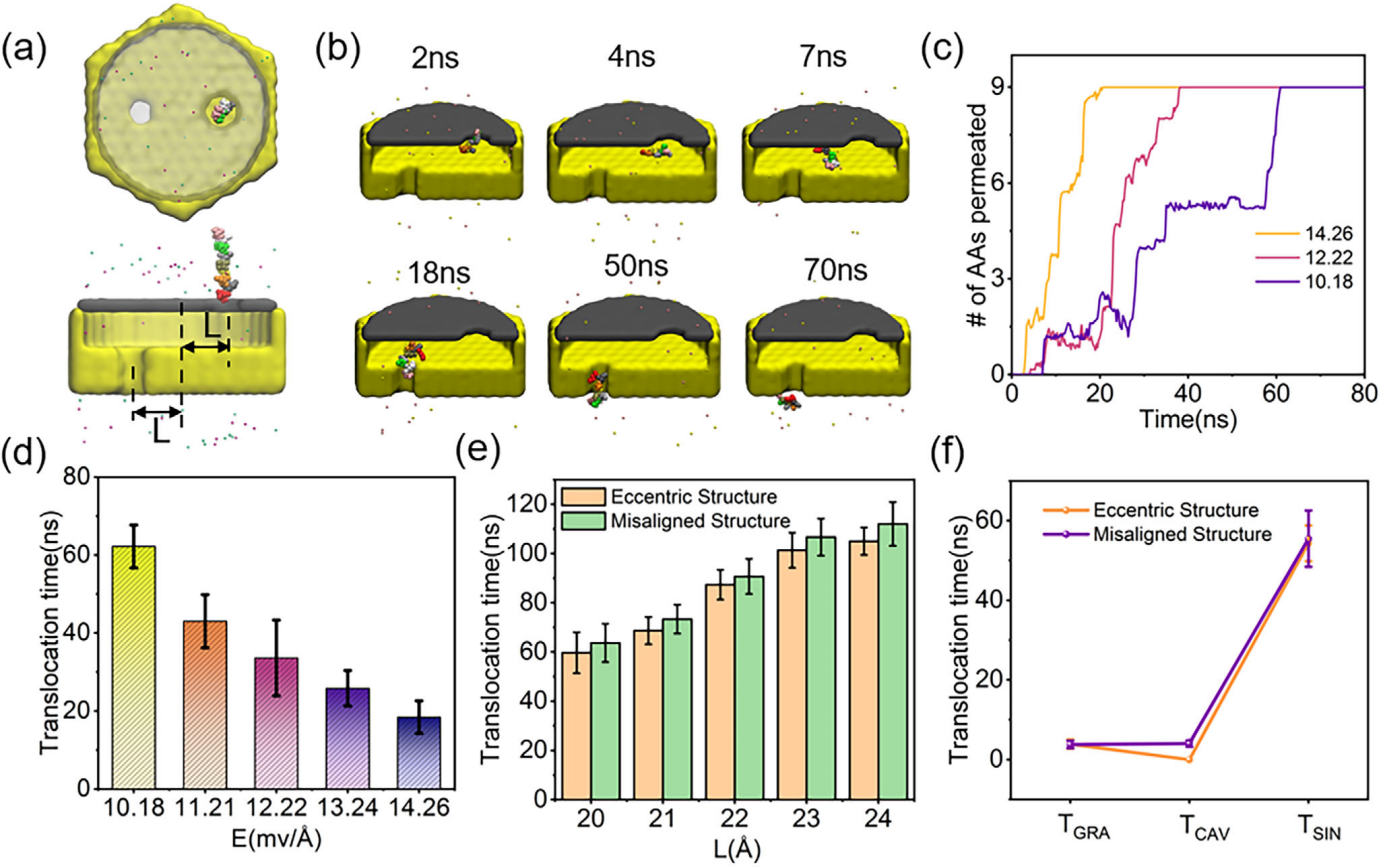
Simulations of peptide translocation through the misaligned nanopores in the graphene/Si_3_N_4_ dual membrane cavity structure. (a) Top view and side view of the simulation system (graphene layer transparent for clarity). The distance from the graphene and Si_3_N_4_ nanopores to the center of the system is L. The coloring method for all the materials in the system is same as that used for Figure 1. (b) A series of microscopic configurations of peptide translocation through the cavity structure vs. time. The peptide moves electrophoretically from *cis* side to *trans* side under an electric field of 10.18 mV/Å (*L*  =  20). (c) The number of protein residues permeated through the Si_3_N_4_ nanopore vs. time (d) The effect of external field strength *E* (mV/Å) on the translocation time of the peptide through the cavity structure. (e) Translocation time of the peptide through the system with different distances *L*. (f) Translocation time comparison for the peptide moving through the graphene nanopore, cavity and Si_3_N_4_ nanopore.

In all three systems described above, it is found that the peptide translocates fast through the cavity channel. To investigate whether it is possible to slow down the peptide translocation inside the cavity channel, we set up another cavity structure that makes the graphene and Si_3_N_4_ nanopores misaligned, and the two nanopores are distributed on both sides of the cavity structure, as shown in Figure 4a. This physical misalignment of the two nanopores forces the peptide to move laterally between the two nanopores to pass through the cavity channel. Figure 4b shows the microscopic configurations of the peptide translocation through the system. The corresponding CoM of peptide is shown in Figure S7. Movie S4 shows the simulation trajectory of the whole process for translocation. The peptide firstly passes through the graphene nanopore, then it moves laterally within the cavity for due to the misalignment of the two nanopores, and finally exits the Si_3_N_4_ nanopore. We also observed that the migration rate increases with the intensity of the external electric field (Figures 4c,d). We then compared the translocation times, as shown in Figure 4e, under an electrical field of 10.18 mV/Å, the time for misaligned structure is only a few nanoseconds longer than that for eccentric structure as the distance *L* increases. Analysis of the translocation time reveals that the total time for peptide translocating through the misaligned nanopore cavity structure consists of the time through the graphene pore, the Si_3_N_4_ nanopore, and the cavity channel. Aside from *T_CAV_*, the times for the other two stages are basically comparable with the former three systems (Figure 4f). Thus, misaligning the two nanopores only increases the translocation time inside the cavity, however the actual translocation velocity through the individual pores (graphene and Si_3_N_4_ nanopores) is not obviously influenced.

**FIGURE 5:**
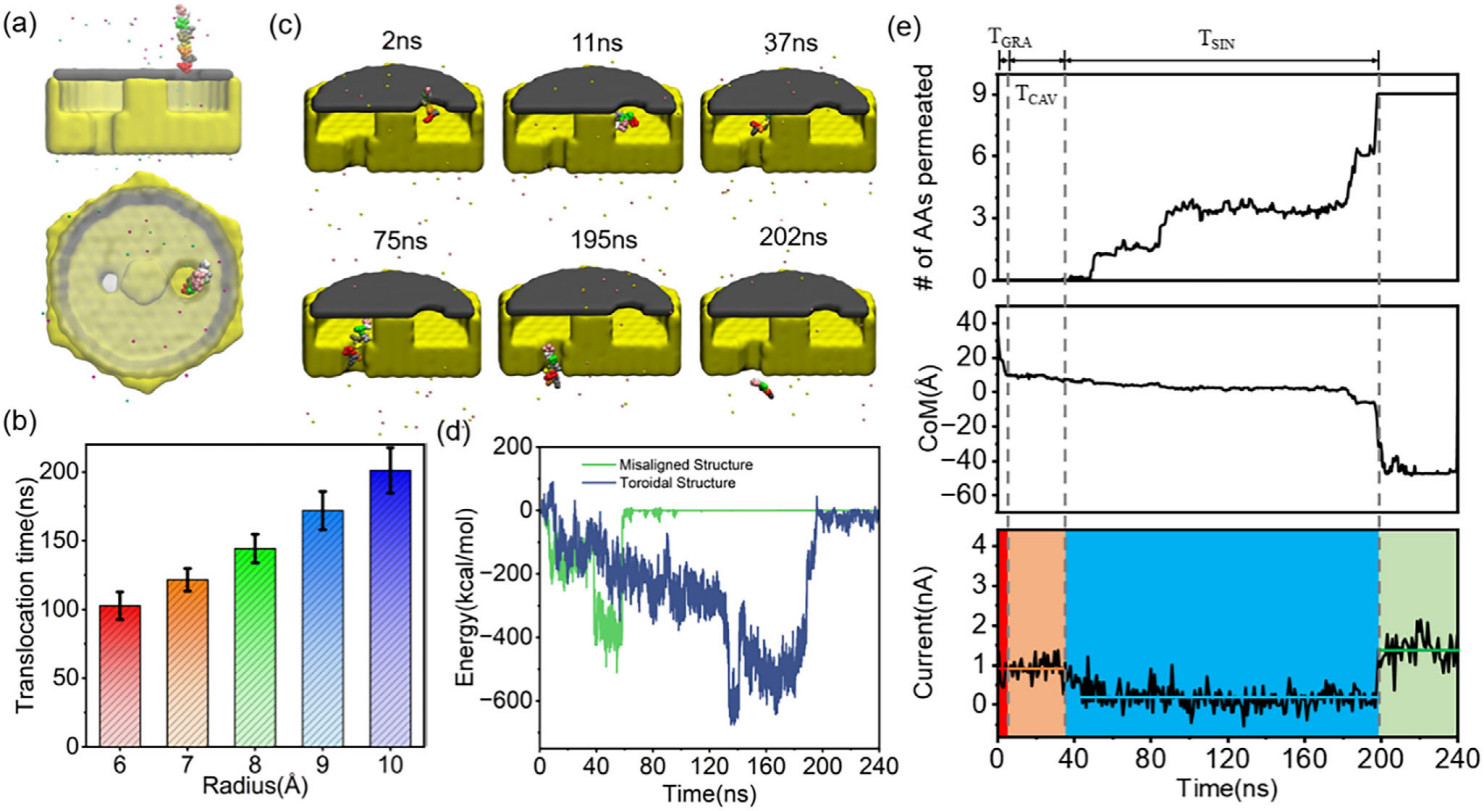
Simulations of peptide translocation through the misaligned nanopores in the graphene/Si_3_N_4_ dual membrane cavity structure with a roundabout located at the middle of the cavity. (a) Side view and top view of the simulation system (graphene layer transparent for clarity). The coloring method for all the other materials in the system is same as that used for Figure 1. (b) The translocation time of the peptide through the cavity structure under different radius of roundabout. (c) A series of microscopic configurations of peptide translocation through the cavity structure vs. time. The peptide moves electrophoretically from the *cis* side to *trans* side of the cavity structure with *R*  =  10 Å of the roundabout. (d) The interaction energy between the peptide and Si_3_N_4_ vs. time. (e) The number of protein residues permeated through the Si_3_N_4_ nanopore, the center of mass position of peptide along z axis and the ionic current trace vs. time during the entire translocation process of peptide through the cavity structure, respectively. Compared to the system shown in Figure 1, this system includes an additional time (shown by the orange region) that is for the peptide moving around the Si_3_N_4_ roundabout.

Based on the above findings, we further proposed the toroidal cavity structure to study whether it is feasible to reduce peptide translocation rate. In this design, a roundabout (radius *R*) is retained within the cavity, forcing the peptide to navigate around a physical obstacle and move closely against the walls while passing through the cavity channel. For simulations of peptide translocation through the misaligned nanopores in the graphene/Si_3_N_4_ dual membrane cavity structure with a roundabout located at the middle of the cavity, the distance (*L*  =  20) between the two nanopores and the center of the system remains constant (Figure 5a). We investigated the effect of this structure on peptide translocation rate by varying the roundabout radius *R* (Figure 5b). Figure 5c shows the microscopic configurations of peptide conformation during its translocation process. Movie S5 shows the simulation trajectory of the whole process for translocation. The peptide firstly passes through the graphene nanopore, then begins to permeate towards the Si_3_N_4_ nanopore while wrapping around the roundabout due to the physical obstruction. It is found that the translocation time increases with radius *R*. For example, when the radius of roundabout reaches 10 Å the toroidal cavity structure extends the translocation time nearly eight times longer than that for the system (central structure) shown in Figure 1. Comparing the other systems described in the work, under identical conditions, the interaction energy in the toroidal cavity is far greater. The interaction energy plotted in Figure 5d shows that the strong interaction between the peptide and Si_3_N_4_ significantly reduces the peptide's translocation rate, proving that the deceleration primarily stems from enhanced van der Waals interactions due to increased interaction area between the peptide and Si_3_N_4_ material. The toroidal structure exerts both steric hindrance and interaction effects, consequently, the translocation rate is substantially reduced not only in the cavity channel but, more importantly, in the critical Si_3_N_4_ nanopore region. The introduction of the toroidal cavity significantly prolongs the duration of current blockade (Figure 5e). Similar results for three other successful peptide translocation cases through the Si_3_N_4_ nanopore are presented in Figure S8. Analysis of *T_GRA_*, *T_CAV_*, and *T_SIN_* shows significant increases in all stages (Figure S9). It is evident that this system achieves a complete deceleration of the peptide migration process by incorporating a toroidal cavity structure at the center. Notably, the specific stages of peptide translocation through the Si_3_N_4_ nanopore reveal a unique stick‐slip dynamic process of peptide, the enhanced vdW forces between the peptide and the cavity walls are strong enough to counteract the electric drive, inducing observable pauses. This quasi‐static behavior greatly extends the signal acquisition window, which is vital for improving temporal resolution. Importantly, the blocking currents generated as the peptide sequentially passes through the graphene nanopore, the cavity, and the Si_3_N_4_ nanopore differ, resulting in a multi‐stage continuous current trace that provides high‐resolution spatial localization of the peptide. In conclusion, the toroidal cavity structure significantly reduces peptide translocation rate, particularly through the critical sensing region, providing a potential solution for high‐resolution protein sequencing based on nanopores.

In this study, by constructing a Graphene/Si_3_N_4_ dual membrane cavity system, a spatial geometry‐driven multi‐level regulation strategy was successfully implemented. This structure achieves hierarchical control over the entire protein translocation process by independently regulating charge capture at the graphene nanopore, geometric movement trajectories within the cavity, and the sensing environment of the Si_3_N_4_ nanopore, thereby overcoming the limitations of traditional single‐membrane structures in controlling translocation speeds. Furthermore, the highly characteristic multi‐level continuous current traces generated by this system exhibit exceptional sensing value, providing high‐resolution spatial localization for the molecule's translocation trajectory. Specifically, the current steps produced by the graphene pore and the cavity can serve as trigger signals (pre‐warning signals) before the peptide enters the critical Si_3_N_4_ sensing region. This finding demonstrates the significant potential of the dual membrane cavity structure in achieving high‐precision, high‐spatial‐resolution protein sensing.

Additionally, to verify the universality of the toroidal cavity structure, this study supplemented simulations for two different peptide sequences by replacing Ala at the fourth position with Leu and Tyr (denoted as EL and EY, respectively, while the original peptide is denoted as EA). Their translocation times and ionic current traces are detailed in Figures S10 and S11. The results show that replacing Ala with Leu or Tyr in the peptide sequence significantly increases the translocation time. By comparing Ala (small side chain), Leu (large hydrophobic side chain), and Tyr (bulky aromatic side chain), it is demonstrated that despite the different physicochemical properties of the side chains, the toroidal cavity structure consistently produces a significant deceleration effect. Due to the relatively larger molecular weights of Leu and Tyr, the larger and more complex side chains lead to stronger adsorption between the peptide and the cavity, further prolonging the translocation time. Moreover, Tyr possesses an aromatic ring structure, resulting in an average current blockade significantly larger than that of Ala. These simulation results confirm the applicability of the dual membrane cavity system to other sequences and its capability to distinguish between different peptide sequences.

We acknowledge the fabrication challenges associated with engineered cavity geometries. However, we have provided a feasible fabrication roadmap and preliminary results. Using Focused Ion Beam (FIB) milling, we have successfully etched a circular cavity and a Si_3_N_4_ nanopore at specific positions on a Si_3_N_4_ membrane (see in Figure S12). For graphene attachment, mechanical exfoliation offers a high‐quality lattice structure preferred for physical research. We successfully transferred graphene onto the Si_3_N_4_ membrane, confirmed by Raman spectroscopy (Figure S13). The complex toroidal cavity can be realized via Electron Beam Lithography (EBL) and Reactive Ion Etching (RIE). The process involves defining an annular exposure area with EBL, followed by RIE to create a toroidal groove surrounding a central pillar. Finally, FIB is used to drill the nanopore. This mature combination of EBL for pattern definition, RIE for depth control, and FIB for precision nanopore drilling provides a technologically feasible pathway.

## 3 Conclusion

This study addresses the issue of excessively translocation rates of proteins in nanopores sequencing by proposing and designing a graphene/Si_3_N_4_ dual membrane cavity system. Through molecular dynamics simulations, we systematically investigated the effects of different structures on the migration behavior of peptide, aiming to identify control strategies that effectively reduce peptide translocation rates without increasing the complexity of the sensing environment. Results indicate that extremely rapid translocation of the peptide through the entire system, in the initial central structure. Through charge regulation, we discovered that electrically charged graphene nanopores can slightly slow down peptide translocation through the graphene nanopore, but fail to reduce translocation rate through the Si_3_N_4_ nanopore. Moreover, when σ  =   −0.128 *mC*/*m* ^2^, the initial electrostatic repulsion energy reached approximately 150 kcal/mol, far exceeding the van der Waals attraction. This resulted in an energy barrier higher than the energy provided by electrophoretic drive, ultimately causing capture failure. However, once a peptide was captured by the graphene nanopore, it was rapidly transported through the Si_3_N_4_ nanopore.

Next, we explored altering nanopore positioning. In the eccentric structure, coaxial dual nanopores were displaced from the center of system. As the distance L increased, the interaction energy between the peptide and Si_3_N_4_ strengthened. This indicates enhanced adsorption of the peptide by Si_3_N_4_, effectively slowing its penetration rate. Consequently, shifting the coaxially aligned nanopores away from the center significantly slow down the peptides translocation rate to traverse the Si_3_N_4_ nanopore. In the misaligned structure, the two nanopores are offset. As the distance L increases, the time for misaligned structure is only a few nanoseconds longer than that for eccentric structure. This misalignment merely increases the translocation time within the cavity, but the actual translocation rate through individual pores (graphene and Si_3_N_4_ nanopores) is not obviously influenced.

Based on the above findings, we further the toroidal cavity structure. This system incorporates a toroidal cavity channel, forcing the peptide to navigate around a physical obstacle and move closely against the walls while passing through the cavity channel. This achieves complete deceleration of peptide migration, with translocation time nearly eight times longer than in the initial central structure. Moreover, the enhanced van der Waals forces between peptides and cavity walls sufficiently counteracted the electric drive, inducing observable pauses. This quasi‐static behavior substantially extended the signal acquisition window, proving crucial for enhancing temporal resolution. In summary, the toroidal cavity structure significantly reduced peptide translocation rate, offering a potential solution for high‐resolution protein sequencing based on nanopores.

This study designed a graphene/ Si_3_N_4_ dual membrane cavity system to investigate peptide translocation processes through multiple strategies, aiming to control peptide translocation rates through nanopores and uncover underlying mechanisms. Although the translocation rate of central structure peptides was initially rapid, successful reduction of protein translocation rate in the Si_3_N_4_ sensing system was achieved through charge regulation and cavity structure modification. This provides theoretical guidance for designing nanopore structures with higher sensitivity and selectivity, offering a feasible approach to meet the high‐bandwidth demands of protein sequencing. We anticipate these findings will advance the future development of nanopore sequencing technology and drive new breakthroughs in biomedical and proteomics research.

## 4 Methods

All simulations in this work were performed using the NAMD 2.14 program with a time step of 2 *f*s [43]. Periodic boundary conditions were applied along the *x*, *y*, and *z* directions. The simulation system constructed in this study consists of Si_3_N_4_ membrane, graphene membrane, peptide molecule, potassium ions (K^+^), chloride ions (Cl^−^), and water molecules. The CHARMM36 force field and CUFIX corrections were used [44, 45], and all graphene atoms were treated as aromatic carbons of atom type CA. The Si_3_N_4_ membrane thickness is 3.4 nm. A cylindrical cavity with a diameter of 8.6 nm is etched into the upper half region of the Si_3_N_4_ membrane, while a Si_3_N_4_ nanopore with a diameter of 1.4 nm is generated in the lower half region. Subsequently, a single‐layer graphene is transferred on the Si_3_N_4_ membrane to form a cavity. A nanopore is also drilled in the graphene membrane to allow ions, water molecules and peptides to permeate through the cavity structure. The sequence of the peptide is EEEAEDLQV. To conserve computational resources and focus on the Si_3_N_4_ nanopore region, the graphene pore diameter was set to 1.7 nm, and the peptide molecule was initially positioned 0.3 nm above the graphene pore to ensure rapid capture. Potassium and chloride ions were added to the system to maintain its neutrality, resulting in a final ion concentration of 1 M. The final system contained approximately 67 000 atoms. Harmonic constraints of 10.0 kcal mol^−^^1^ Å^−^^2^ were applied to all atoms on the Si_3_N_4_ membrane to constrain them at their initial positions. To prevent the protein molecule from drifting away from the nanopore during minimization and equilibration, a harmonic constraint of 1.0 kcal mol^−^^1^ Å^−^^2^ was applied to the protein. The system temperature was maintained at 295 K using a Langevin thermostat applied to all heavy atoms [46, 47], with a damping coefficient of 1 ps^−^^1^. The RATTLE [48] and SETTLE [49] algorithms were utilized to handle covalent bonds involving hydrogen atoms in proteins and water molecules, respectively. The particle mesh Ewald (PME) algorithm was utilized to calculate the long‐range electrostatic interaction over a 1 Å‐spaced grid [50]. The system underwent energy minimization for 9,600 steps using the conjugate gradient method, followed by equilibration for 24 ns under constant number of atoms, pressure, and temperature (NPT) ensemble. During the simulation, the protein molecule was fully released to allow successful electrophoretic translocation through the nanopore. Production simulations under external electric field strength were performed under constant number of atoms, volume, and temperature (NVT) ensemble. Instantaneous ionic current was calculated by dividing the sum of ionic displacements between consecutive frames of the simulation trajectory by the time interval. Visual Molecular Dynamics (VMD) and Tcl scripts were used to view and analyze all simulation trajectories. All data were sampled at 2.4 ps intervals. Ionic currents were block‐averaged in 0.24 ns units. Each condition was replicated at least three times. All data are expressed as mean ± standard error. Ionic currents were calculated using the formulas below:

(1) $$I\left(t\right)=\frac{1}{\delta tL_{z}}\sum_{j=1}^{N}q_{j}\delta z_{j}\left(t\right)$$

(2) $$\delta z_{j}\left(t\right)=\left\{\begin{array}{c} z_{j}\left(t+\delta t\right)-z_{j}\left(t\right),\left|z_{j}\left(t+\delta t\right)-z_{j}\left(t\right)\right|<L_{z}/2\\ z_{j}\left(t+\delta t\right)-z_{j}\left(t\right)+L_{z},z_{j}\left(t+\delta t\right)-z_{j}\left(t\right)<-L_{z}/2\\ z_{j}\left(t+\delta t\right)-z_{j}\left(t\right)-L_{z},z_{j}\left(t+\delta t\right)-z_{j}\left(t\right)>L_{z}/2 \end{array}\right.$$

where δ*t* = 2.4 ps, *L*_z_ is the length of the system in the *z* direction, *N* is the number of ions, *q*_j_ and *z*_j_ are the charge and *z* coordinate of the *j*th atom, respectively. To reduce the uncertainty of the current calculation, the equation runs over all ions.

A total of five different model systems were constructed, denoted as coaxially aligned nanopores at the center of the graphene/Si_3_N_4_ dual membrane cavity structure (center structure), coaxially aligned nanopores at the center of the graphene/Si_3_N_4_ dual membrane cavity structure with charged graphene nanopore (charge regulation), coaxially aligned nanopores displaced from the center of the graphene/Si_3_N_4_ dual membrane cavity structure (eccentric structure), misaligned nanopores in the graphene/Si_3_N_4_ dual membrane cavity structure (misaligned structure) and misaligned nanopores in the graphene/Si_3_N_4_ dual membrane cavity structure with a roundabout located at the middle of the cavity (toroidal structure). Comparing results obtained from these five cavity structures, it allows for a progressive understanding of structural design laws regarding entrance charge regulation, geometric pore offset, and cavity morphology engineering to slow down the translocation rate of peptide in the Si_3_N_4_ nanopore without complicating the sensing environment.

## Author Contributions

**Yubin Cao**: methodology, formal analysis, visualization, writing – original draft, writing – review and editing. **Junzhou He**: software, visualization, validation. **Wei Si**: supervision, funding acquisition, resources, formal analysis, and conceptualization.

## Conflicts of Interest

The authors declare no conflicts of interest.

## Supporting information




**Supporting File 1**: advs76099‐sup‐0001‐SuppMat.docx.

**Supporting File 2**: advs76099‐sup‐0002‐MovieS1.mp4.

**Supporting File 3**: advs76099‐sup‐0003‐MovieS2.mp4.

**Supporting File 4**: advs76099‐sup‐0004‐MovieS3.mp4.

**Supporting File 5**: advs76099‐sup‐0005‐MovieS4.mp4.

**Supporting File 6**: advs76099‐sup‐0006‐MovieS5.mp4.

## Data Availability

The data that support the findings of this study are available on request from the corresponding author. The data are not publicly available due to privacy or ethical restrictions.
