# Supplementary material for: Nanopore‐Based Protein Deceleration and Sensing Using Graphene/Si3N4 Dual Membrane Cavity
Source: Adv Sci (Weinh). 2026 Jun 11;13(50):e76099. doi: 10.1002/advs.76099 (PMC13337068; doi:10.1002/advs.76099)
Supplement: Supplementary file 1 [file ADVS-13-e76099-s006.docx]

**Supplementary Information**

**Nanopore-based Protein Deceleration and Sensing using Graphene/Si_3_N_4_ Dual Membrane Cavity**

Yubin Cao^1^, Junzhou He^1^, Wei Si^1^**^*^**

^1^Jiangsu Key Laboratory for Design and Manufacturing of Precision Medicine Equipment, School of Mechanical Engineering, Southeast University, Nanjing 211100, China

*****The correspondence should be addressed to wei.si@seu.edu.cn

Figure S1. Effect of external electric field strength E (mV/Å) on the translocation time of peptides through the system. The average translocation time is derived from four independent simulations, with error bars indicating standard deviation.

Figure S2. Independent molecular dynamics simulation of peptides translocation through the coaxially aligned nanopores at the center of the graphene/Si_3_N_4_ dual membrane cavity structure. (a) The number of permeated residues, center of mass position (along z axis) and ionic current trace versus time are shown in one column from top to bottom, respectively. The number of permeated residues, center of mass position and ionic current trace were all sampled at 2.4 ps intervals, and the ionic currents were also block averaged in 0.24 ns blocks. (b) Same description as above. (c) Same description as above.

Figure S3. Independent molecular dynamics simulations of peptides translocation through the coaxially aligned nanopores at the center of the graphene/Si_3_N_4_ dual membrane cavity structure with charged graphene nanopore (surface charge density of $-0.016mC/m^{2}$). (a) The number of permeated residues, center of mass position (along z axis) and ionic current trace versus time are shown in one column from top to bottom, respectively. The number of permeated residues, center of mass position and ionic current trace were all sampled at 2.4 ps intervals, and the ionic currents were also block averaged in 0.24 ns blocks. (b) Same description as above. (c) Same description as above.

Figure S4. Simulations of peptide translocation through the coaxially aligned nanopores at the center of the graphene/Si_3_N_4_ dual membrane cavity structure with charged graphene nanopore. The time required for peptide translocation through graphene nanopores, cavity channels, and silicon nitride nanopores, respectively, under different charge density of graphene nanopores.

Figure S5. Simulations of peptide translocation through the coaxially aligned nanopores displaced from the center of the graphene/Si_3_N_4_ dual membrane cavity structure. The number of permeated residues versus time at different distances L.

Figure S6. Independent molecular dynamics simulations of peptide translocation through the coaxially aligned nanopores displaced from the center of the graphene/Si_3_N_4_ dual membrane cavity structure. (a) The Van der Waals interaction energy of the peptide with the Si_3_N_4_ nanopore versus time are shown in one column from top to bottom, respectively. (b) Same description as above. (c) Same description as above.

Figure S7. Simulations of peptide translocation through the misaligned nanopores in the graphene/Si_3_N_4_ dual membrane cavity structure. The corresponding CoM of peptide in Figure 4b.

Figure S8. Independent molecular dynamics simulations of peptide translocation through the misaligned nanopores in the graphene/Si_3_N_4_ dual membrane cavity structure with a roundabout located at the middle of the cavity. (a) The number of permeated residues, center of mass position (along z axis) and ionic current trace versus time are shown in one column from top to bottom, respectively. The number of permeated residues, center of mass position and ionic current trace were all sampled at 2.4 ps intervals, and the ionic currents were also block averaged in 0.24 ns blocks. R=6. (b) Same description as above. R=8. (c) Same description as above. R=10.

Figure S9. Independent molecular dynamics simulations of peptide translocation through the misaligned nanopores in the graphene/Si_3_N_4_ dual membrane cavity structure with a roundabout located at the middle of the cavity. (a) The time required for peptide translocation through graphene nanopores, cavity channels, and silicon nitride nanopores, respectively. R=6. (b) Same description as above，R=8. (c) Same description as above, R=10.

Figure S10. The effect of external field strength *E* (mV/Å) on the translocation time of the peptide with different sequences through the misaligned nanopores in the graphene/Si_3_N_4_ dual membrane cavity structure with a roundabout located at the middle of the cavity (R=10). The average translocation time for each was averaged over at least three independent simulations. Error bars represent standard errors.

Figure S11. The current of the peptide through the Si_3_N_4_ nanopore in the graphene/Si_3_N_4_ dual membrane cavity structure with a roundabout located at the middle of the cavity (R=10). The time interval is taken from 80 ns to 180 ns, representing the period during which the peptide is translocating through the Si_3_N_4_ nanopore; the dashed line represents the average current value. (a) Peptide is EY; (b) Peptide is EA.

Figure S12. The Si_3_N_4_ membrane cavity structure. A cavity with a diameter of 2 μm and a depth of approximately 60 nm was first etched on the Si_3_N_4_ membrane using a focused ion beam. Then, a silicon nitride nanopore with a diameter of approximately 40 nm was fabricated within the cavity. The darkest region indicates that the nanopore has fully penetrated through.

Figure S13. Raman spectrum. After transferring the graphene onto the Si_3_N_4_ membrane using mechanical exfoliation, the sample surface was tested using a 532 nm laser. The sample exhibits a distinct G peak at 1553.59 cm⁻¹ and a 2D (G') peak at 2767.72 cm⁻¹, indicating that the sample possesses the typical Raman characteristics of graphene.
